# Physicochemical Changes of Deep-Fat-Fried Chicken Drumsticks Treated with Quercetin-in-Edible Coating during Storage Time

**DOI:** 10.3390/foods10020467

**Published:** 2021-02-20

**Authors:** Kelvin Adrah, Daniel Ananey-Obiri, Reza Tahergorabi

**Affiliations:** 1 Food and Nutritional Sciences Program, North Carolina Agricultural and Technical State University, Greensboro, NC 27411, USA; kadrah@aggies.ncat.edu; 2 Department of Computational Sciences and Engineering, College of Engineering, North Carolina Agricultural and Technical State University, Greensboro, NC 27411, USA; dananeyobiri@aggies.ncat.edu

**Keywords:** quercetin, edible coating, deep-fry, chicken, oxidation, quality

## Abstract

In this study, 10% of chicken protein isolate (CPI) and quercetin (1 mg/mL) were used to develop an edible coating to improve the oxidative stability of deep-fat-fried chicken drumsticks during refrigerated storage (4 °C) for 10 days. Chicken samples with edible coating formulated with only 10% CPI served as the control. Although the thiobarbituric acid reactive substances (TBARS) values of the treated samples were lower than the control samples, no significant differences were observed. Quercetin-treated samples were generally harder than control samples. The pH was reduced by quercetin incorporation (*p* < 0.05). L* and b* values increased, while there was no significant variation in a* values during storage (*p* > 0.05).

## 1. Introduction

Several studies evidenced that oils used in deep-fat frying are absorbed by the food and increase the total fat content [1,2,3]. Consequently, overconsumption of fried foods may lead to obesity, type 2 diabetes, and cardiovascular disease, to name a few. Cardiovascular disease represents the leading cause of death and it is estimated that more than 23.6 million people will die from this by 2030 [4].

About two decades ago, it was suggested that an edible coating is capable of reducing moisture loss and further reducing fat uptake in fried foods [5]. The edible coating can be prepared from different materials or by combining several compounds which are formed on the surface of food products. However, protein-based films gained interest due to their superior mechanical properties. Our previous research showed that the application of an edible coating prepared from protein recovered from fish and chicken processing byproducts results in a significant reduction in fat uptake in deep-fat-fried products [6,7].

Although fat absorbed by fried foods can be significantly reduced by the application of edible coatings, a major contributory factor to the deterioration of meat quality is oxidation, a current challenge to the food industry [8]. Several chemical changes occur once oil is exposed to heat. These include oxidation and polymerization of fatty acids and degradation of triglyceride molecules into free fatty acids and glycerol. This can be exacerbated in the presence of air and water from food (which facilitates the hydrolysis) [9]. Thus, lipid oxidation could lead to shelf-life reduction and the formation of compounds in fried foods that are harmful to human health. Researchers [10] demonstrated that antioxidants can be used to improve the shelf-life of meat products. However, the use of synthetic antioxidants for meat preservation raised consumer concerns due to their toxicological effects and adverse effects on human health [11,12]. In light of these reasons, the need for naturally sourced antioxidants is being prioritized to replace synthetic antioxidants.

Quercetin (3,3′,4′,5,7-pentahydroxyflavone) is a flavonol-type flavonoid ubiquitously present in several fruits and vegetables, such as onions, propolis, cherries, broccoli, and even teas and wines [13,14,15,16]. A significant health benefit of quercetin is its ability to scavenge highly reactive species, such as peroxynitrite and hydroxyl radicals [17]. As an antioxidant, quercetin prevents oxidation by either hindering the formation of reactive oxygen species (ROS) or scavenging species responsible for oxidation initiation (O_2_•^−^,^1^O_2_, etc.) or intercept radical oxidation propagators (LOO•). However, it should be kept in mind that antioxidants act via mixed mechanisms combining different types of antioxidation [18]. Quercetin-3-O-glucoside (Q3G) esters are effective in inhibiting primary oxidation by 50% to 100% in oil-in-water emulsion, and Q3G demonstrated more than 50% primary inhibition in bulk oil [19]. Quercetin is also used to treat hyperlipidemia and the prevention of obesity. In a recent study, obese human subjects were treated with quercetin supplements, and their results showed significant improvement in body composition of overweight and obese subjects [20].

According to a recent study, edible coatings can serve as a medium for incorporating antioxidant agents [21]. The incorporation of antioxidants into edible coatings is a developing technology to promote the development of functional foods, where bioactive compounds are embedded in the edible coating matrix. Bioactive edible coatings have the function of regulating the release rate of bioactive compounds into the desired product, eventually improving the shelf stability of the food product, and consequently impacting the consumer’s health [22,23].

Dark muscles (e.g., chicken drumsticks) contain higher heme protein and inorganic iron content than light muscles (e.g., chicken breast), which are the catalysts for lipid and protein oxidation. Thus, in this research, chicken drumsticks were used to study the oxidation behavior of the dark muscle due to exposure to heat (deep-frying) and quercetin-in-edible coating as an antioxidant compound. The main objective of the present work was to evaluate the potential of a chicken protein-based coating formulated with quercetin in improving the oxidative stability of deep-fat-fried chicken drumsticks.

## 2. Materials and Methods

### 2.1. Chicken Sample Preparation

Chicken drumsticks bought from a local grocery shop were used for the study. Samples were cut using a manually operated cutting device into equal pieces, weighing approximately 10 ± 1 g. Subsequently, the samples were kept in a refrigerator at 4 °C for 24 h.

### 2.2. Protein Isolation from Chicken By-Product

#### 2.2.1. Recovery and Brine Washing of Chicken By-Product

Chicken byproducts, such as skins and meat left on the bone from the chicken drumstick, were minced through a 0.5 cm meat grinder (LEM grinder, 5 Big Bite Grinder-0.35 HP, West Chester, OH, USA). The ground product was washed based on the process described by [24] to reduce the fat content of the protein used for isolation. However, slight modifications were made to the procedure. Next, 1 part of ground chicken was homogenized with 5 parts NaCl with 0.05 M cold NaCl (2–4 °C) for 2 min at 13,000 rpm using a laboratory homogenizer (Homogenizer, OMNI International, Kennesaw, GA, USA). The homogenized sample was centrifuged at a speed of 5000× *g* using a centrifuge (Thermo Scientific, Model ST 16 Centrifuge Series, Asheville, NC, USA) set at 4 °C. The chicken slurry obtained after centrifugation was used for protein isolation.

#### 2.2.2. Isoelectric Solubilization/Precipitation and Quantification of Protein Content

Protein recovery from chicken byproducts was performed according to [25]. The collected chicken slurry was homogenized with cold deionized water (1 part of washed chicken slurry to 6 parts of deionized water) for 5 min at a speed of 13,000 rpm at a controlled temperature of 4 °C. About 6 L of the homogenate was measured into a beaker, and its pH was adjusted to 11.50 ± 0.05 with a 10 N NaOH solution. The solution was held at this pH for 10 min and centrifuged for 20 min at 5000× *g* and 4 °C. Following centrifugation, the solution was separated into three layers. The pH of the middle layer containing the chicken protein was adjusted to 5.5 ± 0.05 to precipitate with 6 N HCl. The solution was left to react for another 10 min and centrifuged for 20 min at 5000× *g* and 4 °C. Two layers were formed after centrifugation. The top layer containing water was discarded and the bottom layer containing the protein, hereafter called chicken protein isolate (CPI), was collected.

The protein content of the CPI was measured using the Bradford method with slight modifications [26]. Bovine serum albumin was dissolved in a solution containing 0.1 M NaOH and 3.5% NaCl to prepare standard solutions. Subsequently, 5 g of CPI was measured and homogenized with 30 mL of 0.1M and 3.5% NaCl using a laboratory homogenizer. The homogenate obtained was centrifuged at 4000× *g* at 4 °C for 30 min. The supernatant was collected for protein analysis. Standard and sample solutions, measuring 20 µL were plated into wells of microplates. Accurately measured 200 µL of Bradford reagent was added into wells that were filled with either standard or sample solutions. The plates were incubated for 20 min at 37 °C. Afterward, samples were placed in a spectrophotometer and read at an absorbance of 595 nm.

### 2.3. DPPH Free-Radical Scavenging Activity of Quercetin

Quercetin dihydrate (>95% purity) was obtained from Selleckchem (Houston, TX, USA). The free-radical scavenging ability of quercetin against DPPH radicals was assessed as described by [27]. Briefly, 100 µL of 0.125, 0.25, 0.5, 1, and 2 mg/mL quercetin was prepared with 70 µM DPPH. Positive and negative control samples of 100 µL butylated hydroxytoluene (BHT) at 10 mM and 100 µL methanol were used, respectively. The samples were incubated for 30 min in the dark at room temperature. Subsequently, the absorbance was measured at 517 nm using a spectrophotometer. The DPPH radical scavenging activity of the tested samples was determined using the following formula:

$$\%\;scavenging\;activity=\frac{absorbance\;of\;quercetin\;or\;BHT\;}{absorbance\;of\;negative\;control}\;\times \;100$$

Further, EC_50_ and T_EC50_ were calculated.

### 2.4. Preparation of Bioactive Edible Coating

Our previous study showed that 10% protein in edible coating resulted in the highest fat uptake reduction in deep-fat-fried chicken drumsticks [7]. Also, EC_50_ was calculated to determine the concentration at which quercetin reduced the DPPH radical by 50% [28]. Thus, edible coatings in this study were prepared by slowly dissolving the CPI in a 1 mg/mL quercetin-in-ethanol solution to form a 10% protein-based edible coating. For control, CPI was formulated with only ethanol to form a 10% edible coating. Glycerol was added at 0.4% (*w*/*w*) of the CPI, and the resulting solution was stirred for approximately 15 min. The pH of the edible coating was adjusted to 11 ± 0.05 with a 10 N NaOH solution. Subsequently, the edible coating was stirred uniformly with a laboratory homogenizer at 13,000 rpm for 1 min. Afterward, the pH was readjusted to 7 ± 0.05 using 6 N HCl solution.

### 2.5. Sample Preparation for Frying

Chicken pieces were rinsed under running water, predusted, and dipped in the edible coating. The coated samples were shaken to remove excess coating. Subsequently, the samples were fully immersed in the batter for 15 s. A commercial batter (Louisiana Chicken Batter Mix, Baton Rouge, LA, USA) was prepared according to the manufacturer’s instructions. Finally, samples were evenly rolled in breadcrumbs.

### 2.6. Deep-Frying

Coated chicken drumsticks were deep-fried at 177.7 °C in a bench-top deep fryer (Presto^®^ Dual ProFryTM/1800W, National Presto Industries Inc., Appleton, WI, USA) for 4 min. Particles of fried food building up in the oil during repeated frying operations dramatically lower the quality of frying oil. To avoid this, new and fresh canola oil was used for each batch of treatment. A data logging thermometer was connected to the fryer to monitor and control the frying temperature. The core temperature was checked to reach 73 °C [29]. The fried samples were removed from the oil using prongs and allowed to cool.

### 2.7. Storage Condition

Fried chicken samples were vacuum packed in polyethylene bags, labeled, and stored for 10 days at 4 ± 1 °C. The samples were randomly taken during the refrigerated storage and tested for the following analyses on days 0, 2, 4, 6, 8, and 10.

### 2.8. TBARS Value

Using the 2-thiobarbituric acid reactive substances (TBARS) assay of malondialdehyde (MDA), the oxidative stability of deep-fat-fried chicken samples was determined according to the previously reported method of [30]. TBARS values were determined by using a molar absorptivity of MDA (156,000 M^−1^ cm^−1^), and results were reported as mg MDA/kg sample.

### 2.9. Color Properties

A portable Minolta Chroma CR-400 colorimeter (Minolta Camera Co. Ltd., Osaka, Japan) was used to evaluate the color profile of chicken drumsticks. The Commission Internationale d’Eclairage of France method was employed to determine the L*a*b* tristimulus color values.

### 2.10. Determination of pH

The deep-fried chicken samples (5 g) were homogenized for 1 min with distilled water (20 mL). The pH value was determined using a calibrated digital pH meter (OMNI International, Kennesaw, GA, USA) [31].

### 2.11. Textural Analysis

Textural properties of samples were assessed using a texture analyzer (Model TA-XT2, Texture Analyzer, Texture Technologies Corp., Scarsdale, NY, USA). A puncture test was done at 5 mm/s using a 1/8 spindle with 15% penetrating thickness of the crust.

### 2.12. Statistical Analysis

For each parameter, a duplicate sample plus three trials were used. Six observations were carried out for consistency of the results. The mean values were expressed as results ± standard deviation. A two-way analysis of variance (ANOVA) was used to determine the effects at a significant level of *p* < 0.05 using SAS system software (SAS, version 16.0, SAS 49 Institute, Cary, NC, USA). The differences in the mean values between treatments were calculated using Tukey’s test.

## 3. Results and Discussion

### 3.1. Antioxidant Value of Quercetin

**Figure 1:**
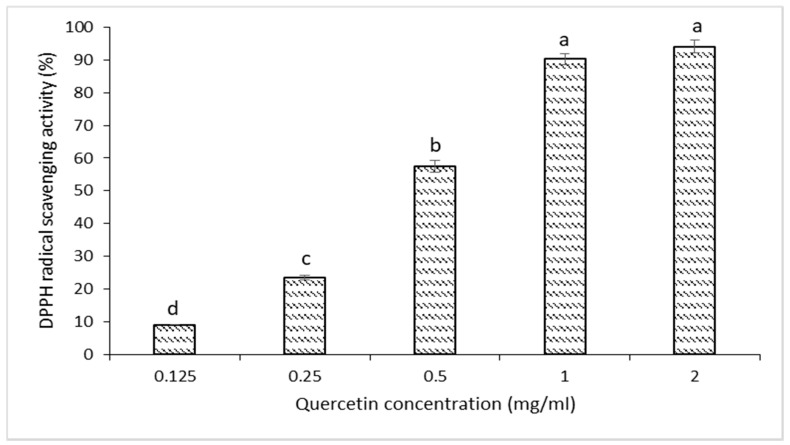
DPPH radical scavenging activity (%) of quercetin. Data are given as means values ± SD. Different letters on the top of data bars indicate significant differences (Tukey’s test, *p* < 0.05) between mean values.

To test the capacity of quercetin to neutralize free radicals, DPPH• (1, 1-diphenyl-2 picrylhydrazyl) scavenging activity assay was conducted. The DPPH radical scavenging activity levels of quercetin at the concentrations of 0.125, 0.25, 0.5, 1, and 2 mg/mL were 9.15, 23.5, 57.57, 90.27, and 94.06%, respectively. As seen in Figure 1, increasing concentration of quercetin reflected a corresponding significantly (*p* < 0.05) higher percentage of inhibition and the DPPH• was reduced to its nonradical form by quercetin. This test showed that quercetin at 1 mg/mL had the highest scavenging activity. This was further confirmed with EC_50_ and T_EC50_ values of 0.4 mg/mL and 4.5 min, respectively. Good antioxidant activity is defined by a low concentration and short time. The research in [32] classified different antioxidant compounds according to T_EC50_ values. Ascorbic acid, alpha-tocopherol, and rutin are classified as rapid, intermediate, and slow in terms of kinetic behavior with T_EC50_ values of 5, 5–30, and >30 min, respectively. Therefore, quercetin could be considered to have rapid antioxidant activity.

### 3.2. Oxidative Stability of Deep-Fat Fried Chicken

**Figure 2:**
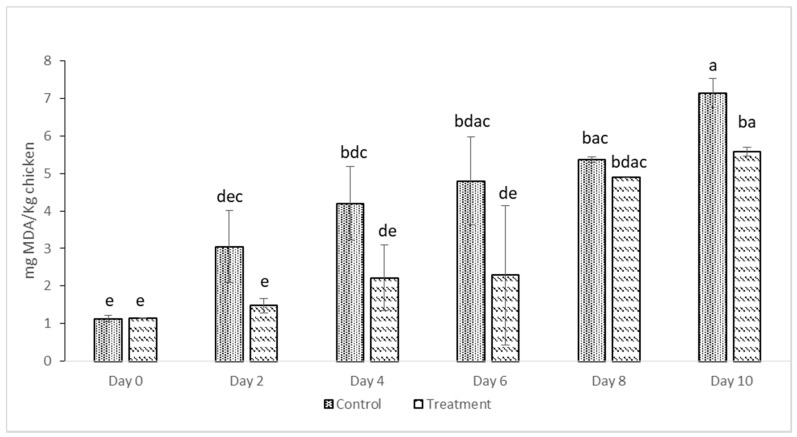
Thiobarbituric acid reactive substances (TBARS) values of control and treated deep-fat-fried chicken drumsticks for 10 days. Data are given as means values ± SD. Different letters on the top of data bars indicate significant differences (Tukey’s test, *p* < 0.05) between mean values.

TBARS was determined to evaluate the efficacy of quercetin in inhibiting undesired changes in fats occurring during the storage of deep-fat-fried chicken samples. The results recorded for malondialdehyde (MDA) contents in control and treated deep-fat-fried chicken samples are shown in Figure 2. The initial TBARS value increased continuously with increasing storage days for control and treated samples from 1.13 mg MDA/kg and 1.15 mg MDA/kg to 7.14 mg MDA/kg and 5.58 mg MDA/kg, respectively. Although the treated samples showed lower numerical values for TBARS, they did not significantly differ from the control samples. A similar ascending trend was found by [33], who measured the TBARS values of emulsion sausage of pork with onion peel extract at 0.05%, since onion peel extract contains quercetin. There was no significant difference between the TBARS values of the control samples, and the samples treated with onion peel extract. However, in contrast to our results, [34] found that when onion peel extract (0.5%) was added to the irradiated pork Jerky, lower peroxide values were recorded. They referred to this effect as the quercetin radical scavenging activity.

A study by [35] showed that when quercetin was mixed with minced fish, the EC_50_ significantly increased and the quercetin lost its efficiency. They assumed this loss of efficiency was due to the interaction between the quercetin molecule and the muscle constituents. However, salt addition did not impact the antioxidant activity of the quercetin.

Furthermore, [36] indicated that boiling would result in an 18% decrease in quercetin concentration compared to raw onion. They noticed a 14.8% and 20.1% decrease in the diglucoside and monoglucoside contents of the boiled onions, respectively, showing the possibility of quercetin leaching into cook water due to low stability.

Others [37,38] showed that five minutes of frying onions in sunflower oil, butter, and rapeseed oil led to 21%, 24%, and 39% quercetin losses. In addition, mechanical peeling of onions during sample preparation in addition to the high temperature of frying could contribute to this quercetin loss [38].

The duration of cooking also appeared to impact on the quercetin loss. Several studies reported that cooking times between 3 and 60 min resulted in 20.6–75% quercetin loss [37,38,39,40,41]. In general, longer cooking time results in greater cooking loss. This loss due to boiling or frying may be due to leaching of the quercetin in the cooking medium rather than chemical degradation of the quercetin. In the case of our study, there might be some leaching of the quercetin while preparing the edible coating. Homogenization may have also contributed as a mechanical factor in further loss of the quercetin in the final product.

Phenolic compounds are capable of limiting oxidation by two major pathways [8]. First, by terminating the oxidation cycle at the propagation phase by giving up electrons to inhibit the formation of lipid and protein radicals, and the second pathway involves eliminating free radicals or reactive oxygen species (ROS) initiators to avoid the actions of chain-initiating radicals. Also, lipid oxidation can be inhibited by stabilizing radical initiators into inactive and insoluble forms. This is achieved by binding metals such as iron and copper as metal chelators. Since quercetin is mainly a phenol-based compound, determination of the radical scavenging activity should be an adequate method to evaluate the antioxidant ability of the quercetin. However, the possibility of lipid and protein interactions with quercetin when blended with the meat is unavoidable, which consequently results in a partial loss in scavenging activity. Further, high temperature during frying and leaching of the quercetin in the edible coating and oil could represent other contributing factors leading to quercetin losses and, consequently, ineffectiveness in lipid oxidation retardation.

### 3.3. Instrumental Color

Data for measured color parameters of deep-fat-fried chicken are shown in Table 1. CIE L* (lightness) values of both control and treated samples decreased gradually toward the end of storage time (*p* < 0.05). Although b* (yellowness) values of the control samples increased when compared with the first day of storage, it remained stable toward the end of storage. The samples treated with quercetin showed fluctuation trends in b* values. A similar result was found for chicken nuggets prepared with leg meat [42]. They related the darker color of the meat to the high-fat content of drumsticks, which resulted in oxidation during storage time and, consequently, higher L* and b* values. Similarly, [43] noticed the same trend of color change for the chicken nuggets during storage time. They justified the color changes in chicken nuggets by myoglobin conversion to metmyoglobin during storage time. Discoloration of meat in response to lipid oxidation is attributed to the formation of aldehydes, alkanes, and conjugated dienes, which are products of the interaction of alkyl and peroxy radicals, which may further cause protein oxidation [8]. However, no significant changes were noticed for a* (redness) values in control and treated samples (*p* > 0.05). A study by [44] found that essential oil addition at high concentrations could result in a reduction of redness due to the prooxidant effect. However, these findings contradict our results, in which no significant changes were observed in a* values.

### 3.4. Texture Properties

**Figure 3:**
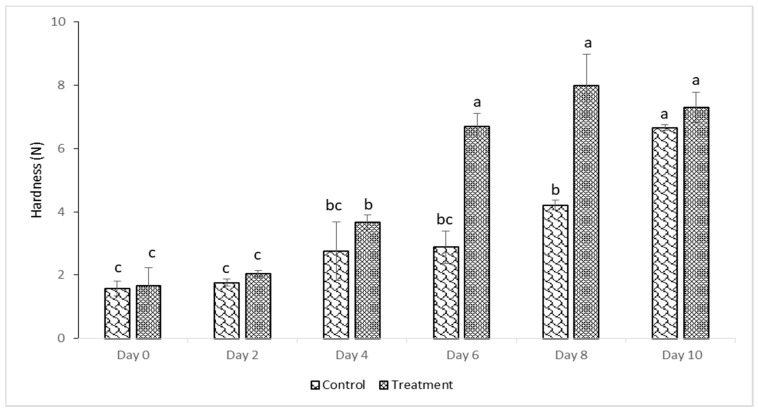
Maximum peak puncture forces of deep-fat fried samples. Data are given as mean values ± standard deviation. Different letters on the top of data bars indicate significant differences (Tukey’s Test, *p* < 0.05) between mean values.

The puncture test mimics the function of the tooth in chewing the food, particularly for crispy, puffed food products. This provides data related to the mechanical properties of the crusts in fried food products which correlates with sensory criteria, i.e., crispiness [45]. Puncture force measured from day 0 up to day 4 of storage revealed no variation between control and treated samples (*p* > 0.05). Puncture force measured on treated samples, however, revealed an increase from day 2 to day 4 and from day 4 to day 6 (*p* < 0.05). Likewise, the force required to penetrate the crust of control samples increased (*p* > 0.05) from day 0 to day 6. As observed in Figure 3, both control and treated samples increased in puncture force during the 10-day storage; nonetheless, it was found that treated samples demonstrated a slightly higher puncture force compared to control samples. These results were consistent with those obtained by [46] who analyzed the textural properties of porcine liver pâté by using penetration tests during refrigerated storage. They noticed significantly higher puncture force values when BHT was added to the liver pâté. Likewise, [47] they also found greater hardness for lamb meat when it was fed on quercetin. The observed increase in puncture results is attributed to the polymerization of lipids and proteins [46]. The lower puncture results in control samples could be due to proteolytic changes that take place in myofibrillar proteins.

### 3.5. pH Value 

**Figure 4:**
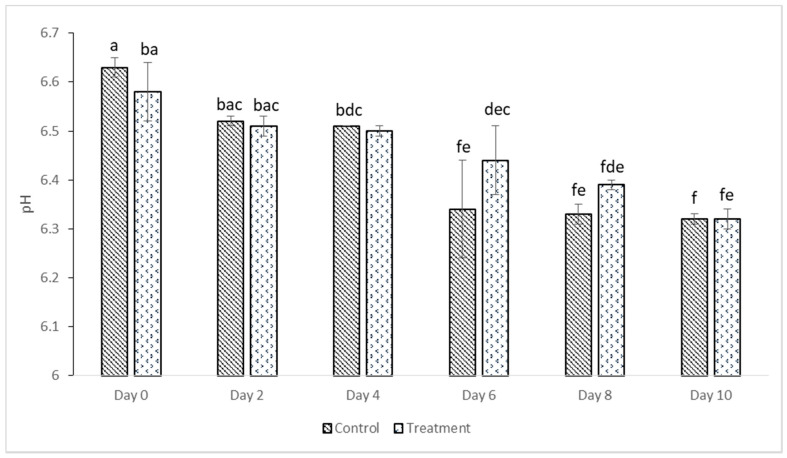
pH values of control and treated deep-fat-fried chicken samples. Data are given as mean values ± standard deviation. Different letters on the top of data bars indicate significant differences (Tukey’s test, *p* < 0.05) between mean values.

Figure 4 shows the effect of quercetin on the pH of deep-fat-fried chicken samples during refrigerated storage at 4 °C for 10 days. The initial pH levels of the control and treatment samples were 6.63 ± 0.02 and 6.58 ± 0.06, respectively. The pH of control samples decreased significantly on day 4 to 6.51 (*p* < 0.05). On the other hand, the pH of treated samples only revealed a statistically significant variation on day 6 (6.44 ± 0.07) (*p* < 0.05). Both control and treatment samples further decreased to 6.32 ± 0.01 and 6.32 ± 0.02, respectively (*p* < 0.05). These results were consistent with findings from [48]. The authors showed that deep-fried chicken nuggets treated with *ganghwayakssuk* ethanolic extract and control decreased in pH until seven days. A vital indicator of food stability associated with microbial and chemical reactions that culminate in food spoilage is the pH [48]. Fernández-López and Sayas-Barberá [49] revealed that the growth of lactic acid bacteria, which results in lactic acid build-up, among other factors, could be attributed to a decrease in the pH of meat products.

## 4. Conclusions

In the present study, quercetin at 1% was incorporated in the edible coating to enhance the oxidative stability and other physicochemical properties of deep-fat-fried chicken drumsticks during 10 days of storage. However, no significant difference in TBARS values was found between the control and treated samples. Puncture test and CIE L* and b* showed an increase when quercetin was incorporated in the edible coating. Also, the pH values of both control and treated samples decreased by the end of storage time. Therefore, higher concentrations of quercetin may be needed for effective inhibition of lipid oxidation.

## Figures and Tables

**Table 1:** Changes in color of deep-fat-fried chicken drumsticks.

Days of Storage	L*		a*		b*	
	Control	Treatment	Control	Treatment	Control	Treatment
0	52.89 ± 3.89 ^a^	52.23 ± 0.53 ^ab^	7.51 ± 0.91 ^a^	8.88 ± 0.52 ^a^	3.05 ± 0.71 ^c^	6.86 ± 3.65 ^bc^
2	48.71 ± 0.92 ^abcd^	50.54 ± 0.97 ^abc^	8.10 ± 0.48 ^a^	8.69 ± 1.45 ^a^	9.25 ± 1.08 ^ab^	9.64 ± 0.99 ^ab^
4	47.92 ± 1.57 ^abcd^	49.36 ± 0.82 ^abc^	8.03 ± 1.26 ^a^	9.33 ± 0.91 ^a^	12.17 ± 3.24 ^ab^	14.31 ± 2.39 ^a^
6	48.51 ± 1.55 ^abcd^	48.40 ± 2.22 ^abcd^	7.89 ± 0.07 ^a^	8.56 ± 2.15 ^a^	12.51 ± 0.33 ^ab^	12.64 ± 3.17 ^ab^
8	46.19 ± 1.15 ^cd^	47.33 ± 2.25 ^bcd^	7.38 ± 0.50 ^a^	7.78 ± 1.24 ^a^	11.17 ± 1.70 ^ab^	13.52 ± 0.90 ^a^
10	45.75 ± 0.98 ^cd^	43.91 ± 1.62 ^d^	7.17 ± 0.56 ^a^	7.36 ± 1.40 ^a^	12.24 ± 1.64 ^ab^	11.47 ± 2.13 ^ab^

Data are given as mean values ± standard deviation. Different letters within the same column indicate significant differences (Tukey’s test, *p* < 0.05) between mean values.

## Data Availability

Data are contained within this article.
